# Attainment of medical student learning outcomes following general practice specialty trainee supervision: a pilot study

**DOI:** 10.3399/BJGPO.2024.0264

**Published:** 2025-09-24

**Authors:** Julie Carson, Anna Frain, Heidi Emery, Edward Tyrrell, Daniel Crowfoot, Gurvinder Sahota, Emma Wilson, Jaspal Taggar

**Affiliations:** 1 Primary Care Education Unit, Centre for Academic Primary Care, University of Nottingham, Nottingham, UK; 2 Graduate Entry Medicine, School of Medicine, University of Nottingham, Nottingham, UK; 3 Derby General Practice Training Scheme, Derby, UK; 4 Nottingham Centre for Public Health and Epidemiology, School of Medicine, University of Nottingham, Nottingham, UK; 5 Nottingham Centre for Evidenced Based Healthcare, Faculty of Medicine and Health Sciences, University of Nottingham, Nottingham, UK

**Keywords:** undergraduate education, postgraduate education, education, general practice, primary health care

## Abstract

**Background:**

Innovative training approaches are needed to address the lack of capacity in primary care for undergraduate medical student clinical placements. Near peer teaching (NPT) by general practice specialty trainees (GPSTs) is one possible solution.

**Aim:**

To compare the attainment of intended learning outcomes (ILOs) for students taught by GPSTs and qualified GPs.

**Design & setting:**

Quantitative pilot study of medical students undertaking clinical training in general practice at the University of Nottingham.

**Method:**

Year 3 GPSTs were trained to supervise first-year graduate-entry medical students undertaking 6 half-day GP visits (2022–2023). Using Likert-scale post-training questionnaires, self-reported attainment of ILOs was compared for supervision provided to students by GPSTs and GPs. Secondary outcomes included student, GPST, and GP views about NPT.

**Results:**

Of 112 medical students, seven were supervised by GPSTs and 105 by GPs. In total, 101 students responded (seven [100%] from GPST supervision; 94 [90%] from GP supervision). There was no significant difference between groups in attainment of seven ILOs with significantly greater attainment for students supervised by GPSTs for receiving feedback (*P* = 0.018) and self-reflection (*P* = 0.015). GPSTs reported improved organisation, communication, feedback skills, and desire for future student supervision. Medical students and GPs reported enthusiasm for future NPT by GPSTs.

**Conclusion:**

Attainment of ILOs during undergraduate GP placements was at least equivalent when students were supervised by GPSTs compared with GPs. GPSTs are an important group for building supervisory capacity for undergraduate education and this study adds confidence in enabling GPST supervision of undergraduates.

## How this fits in

Despite the growing literature supporting the value of near peer training (NPT) in GP training of undergraduates, there are no studies that have investigated the ability of general practice specialty trainees (GPSTs) to successfully deliver intended learning outcomes (ILOs) from their teaching. This novel study demonstrates attainment of ILOs during undergraduate GP placements was at least equivalent from GPST supervision compared with GPs. The NHS Long-Term Workforce Plan aims to increase medical school and GP training posts, which will require increased supervisory capacity for student placements. These findings give confidence regarding GPST supervision of undergraduates.

## Introduction

General practice is under unprecedented pressures.^1^ Increasing patient demand, greater multimorbidity, and complex patient needs, combined with 1790 fewer full-time equivalent GPs than in 2015 are identified as significant drivers for these pressures.^2^ This has now translated into education and workforce training sectors. Substantial challenges are reported for sustaining the capacity of training healthcare professionals in general practice.^3^ These difficulties are set to rise in parallel to implementing the NHS Long-Term Workforce Plan (LTWP), which aims to significantly increase medical school and GP training posts.^4^ This inevitably will require an increased supervisory capacity for student placements in general practice.

The Health Education England (HEE) and the Medical Schools Council (MSC) commissioned report, *By Choice, Not By Chance*, and systematic evidence syntheses advocate for increasing medical student choices in GP careers through greater exposure in primary care to positive and enthusiastic role modelling during clinical training.^5,6^

There has been a growing emphasis on the importance of role modelling through near peer teaching (NPT) in general practice. Although there is no universal definition of NPT, it is generally agreed to be a form of peer education in which the teacher has participated in the same training as the student but is at least one academic year ahead in their professional journey.^7^ In general practice, the term NPT can refer to medical student training delivered by GP specialty trainees (GPSTs). GPSTs are more likely to be cognitively and socially congruent with medical students leading to teaching that better meets learner needs in more relaxed teaching environments.^8^ Despite the growing emphasis for the utility of NPT in general practice, the research underpinning this training modality has limitations and, consequently, NPT is routinely adopted only in hospital settings^9–11^

Most studies investigating NPT of medical students by GPSTs have been qualitative with small sample sizes but have identified positive outcomes from NPT for the stakeholders involved. A qualitative study of GPSTs teaching medical students during their training posts found medical students valued the teaching received and GPSTs benefited from additional professional development.^12^ A review of literature similarly identified the benefits of NPT for both learners and teachers but also identified a number of barriers to its implementation.^13^ In particular, GPs and GP trainers lacked confidence in the competencies of GPSTs to deliver training. While teachers have a multitude of roles and benefits to students during an educational journey, one fundamental outcome from teaching is to enable the attainment of learning outcomes from education delivered.^14^ Despite the growing literature supporting the value of NPT in GP training of undergraduates, there are no studies that have investigated the ability of GPSTs to successfully deliver intended learning outcomes (ILOs) from their teaching.

Therefore, this pilot study aimed to compare the attainment of ILOs from medical student training when delivered by GPSTs through NPT and GPs during undergraduate medical training.

## Method

### Setting

Graduate Entry Medicine (GEM) students at Nottingham Medical School undertake early clinical experience (ECE) placements in their first year of study, comprising 6 half-day visits to the same general practice across a 1-year period. Students are usually supervised by qualified GPs known as GP tutors who receive tutor training to supervise students and deliver teaching to attain ILOs.

Students experience a variety of consultations aiming to embed classroom learnt communication and examination skills within live clinical environments. Each visit occurs towards the end of a classroom problem-based learning (PBL) module. During each GP visit medical students record their experiences from a minimum of six patient contacts.

Between August 2022 and June 2023, a pilot study was undertaken for GEM students to be supervised by GPSTs rather than GP tutors.

### Academic context

Course learning materials and resources were shared with all GP tutors and GPSTs taking part. The GPSTs were required to undertake training that was similar to that provided to GP tutors. This comprised the following:

- 3-hour training session covering teaching and feedback skills before the first visit;
- 1-hour debrief session after the first visit; and
- Individual support, which was available from the university faculty throughout the academic year, if requested.

All GEM students received an educational guidebook detailing the configuration of GP visits and nine ILOs for the 1-year GEM visits:

1. Help illustrate the significance of basic medical sciences to clinical settings
2. Allow students to practise communication skills
3. Allow students to perform clinical examinations
4. Promote discussion of personal and professional development
5. Encourage understanding of the roles of different professionals involved in patient care
6. Help students develop the ability to evaluate the ethical and legal issues in patient care
7. Provide feedback on performance and encourage self-reflection
8. Promote skills of self-evaluation and appraisal leading to reflective medical practice
9. Stimulate and maintain students’ enthusiasm for clinical medicine early in the course

### Recruitment

Third-year GPSTs of the Derby GP Specialty Training Scheme were recruited for the study as the GEM visits were scheduled to occur during year 3 of specialty training (ST3). However, the project was first introduced to all GPSTs at the end of specialty training year 2 (ST2) to allow recruitment and training before the first visit.

GPST participation was voluntary (opt-in). Medical students were randomly allocated with either GPST or GP tutor. After allocation, students had the option to move to a GP supervisor if requested (opt-out). GPSTs received a maximum of one medical student to supervise.

### Data collection

Evaluation of the project was undertaken using surveys to determine the attainment of ILOs by medical students and stakeholder views of NPT of GEM students.

Four surveys were distributed:

1. Medical student survey. This was distributed to all medical students after completing all six visits. Questions included age (categorised:<25 years; 25–30 years; 31–35 years; 36–40 years; 41–45; not stated), gender, and self-assessed attainment of the nine ILOs rated by a 5-point Likert scale. Free-text questions ascertained views about their experiences. See Supplementary Survey 1.
2. GPST survey A. This was distributed to all ST2 GPSTs who were notified about the project. The survey was completed pre-visits to ascertain motivation for participation (or not) using tick-box options and free-text questions. See Supplementary Survey 2.
3. GPST survey B. This was distributed to all ST3 GPSTs who took part in the project and after all six visits were completed. Questions ascertained age, gender, and six outcomes from NPT were scored using a 5-point Likert scale (communication skills development; organisation skills development; development of skills in giving feedback; future desire to supervise students; future desire to be a GP trainer; receiving support from GP trainer). Free-text questions ascertained information about overall experience. See Supplementary Survey 3.
4. GP trainer survey. This was distributed to the GP trainers of the participating GPSTs after medical students completed all six visits. Questions included length of time as a trainer, views on the project via a 5-point Likert-type items as previously and free-text questions about the experience. See Supplementary Survey 4.

Likert scales rated participant responses as: strongly agree; agree; neither agree nor disagree; disagree; or strongly disagree.

The attainment of ILOs was defined and represented by categorising all strongly agree and agree responses by medical students when reporting ILO attainment. All responses of neither agree nor disagree; disagree; strongly disagree for attainment of ILOs were used to define non-attainment.

### Analyses

Data were summarised as numbers and percentages for the categorical data. Comparisons across groups were made using the χ^2^ test for categorical data and Fisher’s exact test where appropriate. A *P*<0.05 was used to determine statistical significant differences across groups and all analyses were undertaken using SPSS (version 29).

Qualitative free-text data were analysed using content analysis to identify emergent stakeholder views for the facilitators and barriers of NPT.^15^

## Results

### Participant response and demographics

The study included 112 medical students enrolled on the GEM programme. Of 18 GPSTs invited, seven (39%) were recruited to the study each supervising one medical student. The remaining 105 medical students were supervised by GP tutors. No students requested transfer back to GP tutor supervision from GPSTs.

Of 112 medical students, 101 (90%) responded to the survey (7/7 [100%] from GPST supervision; 94/105 [90%] from GP tutor supervision). Seven out of seven (100%) of GPSTs and 2/7 (29%) GP trainers of the GPSTs completed their respective surveys. Of the 18 GPSTs invited to participate, 18 (100%) responded to the pre-study survey providing data about motivation for NPT.

Of the 101 responders, 64 (63%) students were female and 81 (80%) were aged <30 years. There were no significant differences in the age and gender distribution of medical students according to supervisor allocation (Table 1).

**Table 1.** Age and gender of medical students stratified by supervisor allocation

	Supervisor *n* (%)		*P*-value^a^
	GP	GPST	
**Gender**			
Male	33 (35%)	2 (29%)	1.0
Female	59 (63%)	5 (71%)	
Prefer not to say	2 (2%)	0 (0%)	
**Age, years**			
<25	47 (50%)	3 (43%)	0.52
25–30	28 (30%)	3 (43%)	
31–35	12 (13%)	0 (0%)	
36–40	5 (5%)	0 (0%)	
41–45	1 (1%)	1 (14%)	
Prefer not to say	1 (1%)	0 (0%)	

**Total**	94	7	

^a^ *P* = significance value by Fisher’s exact test. GPST = general practice specialty trainees

### Medical student attainment of ILOs

Outcomes for the attainment ILOs of medical students supervised by GPSTs and GP tutors are provided in Table 2.

**Table 2.** Comparison of the attainment of ILOs from GPST and GP tutor supervision

Intended learning outcome	Supervisor	Non-attainment	Attainment	*P*-value^a^
The visits helped me understand the importance and relevance of basic medical sciences within a clinical setting	GP	2 (2%)	92 (98%)	0.20
	GPST	1 (14%)	6 (86%)	
I had opportunities to practise my communication skills	GP	40 (43%)	54 (57%)	0.70
	GPST	2 (29%)	5 (71%)	
I had opportunities to perform clinical examinations	GP	41 (44%)	53 (56%)	0.23
	GPST	1 (14%)	6 (86%)	
I was able to discuss my personal and professional development with my placement supervisor	GP	31 (33%)	63 (67%)	0.097
	GPST	0 (0%)	7 (100%)	
I gained a better understanding of the roles of different healthcare professionals involved in delivering care at the GP practice	GP	18 (19%)	76 (81%)	1.00
	GPST	1 (14%)	6 (86%)	
I had opportunities to discuss ethical and legal issues that were encountered during the placement with my supervisor	GP	19 (20%)	75 (80%)	0.34
	GPST	0 (0%)	7 (100%)	
I was given feedback about my performance by my supervisor	GP	44 (47%)	50 (53%)	0.018
	GPST	0 (0%)	7 (100%)	
I was encouraged to evaluate and reflect on my own performance by my supervisor	GP	46 (49%)	48 (51%)	0.015
	GPST	0 (0%)	7 (100%)	
As a result of my GP visits, I have a greater enthusiasm for clinical medicine	GP	12 (13%)	82 (87%)	0.25
	GPST	2 (29%)	5 (71%)	

^a^ *P* = significance value by Fisher’s exact test. GPST = general practice specialty trainees

Of the nine ILOs, there were no significant differences in student attainment between GPSTs and GP tutor supervision for seven outcomes. Significantly, more medical students reported receiving feedback about their performance by GPSTs than GP tutors (7 [100%] versus 50 [53%]; *P* = 0.018). Also, significantly more students reported being encouraged to evaluate and reflect on their performance by GPSTs (7 [100%] versus 48 [51%]; *P* = 0.015].

### Motivation of GPSTs to undertake NPT of medical students

**Figure 1.**
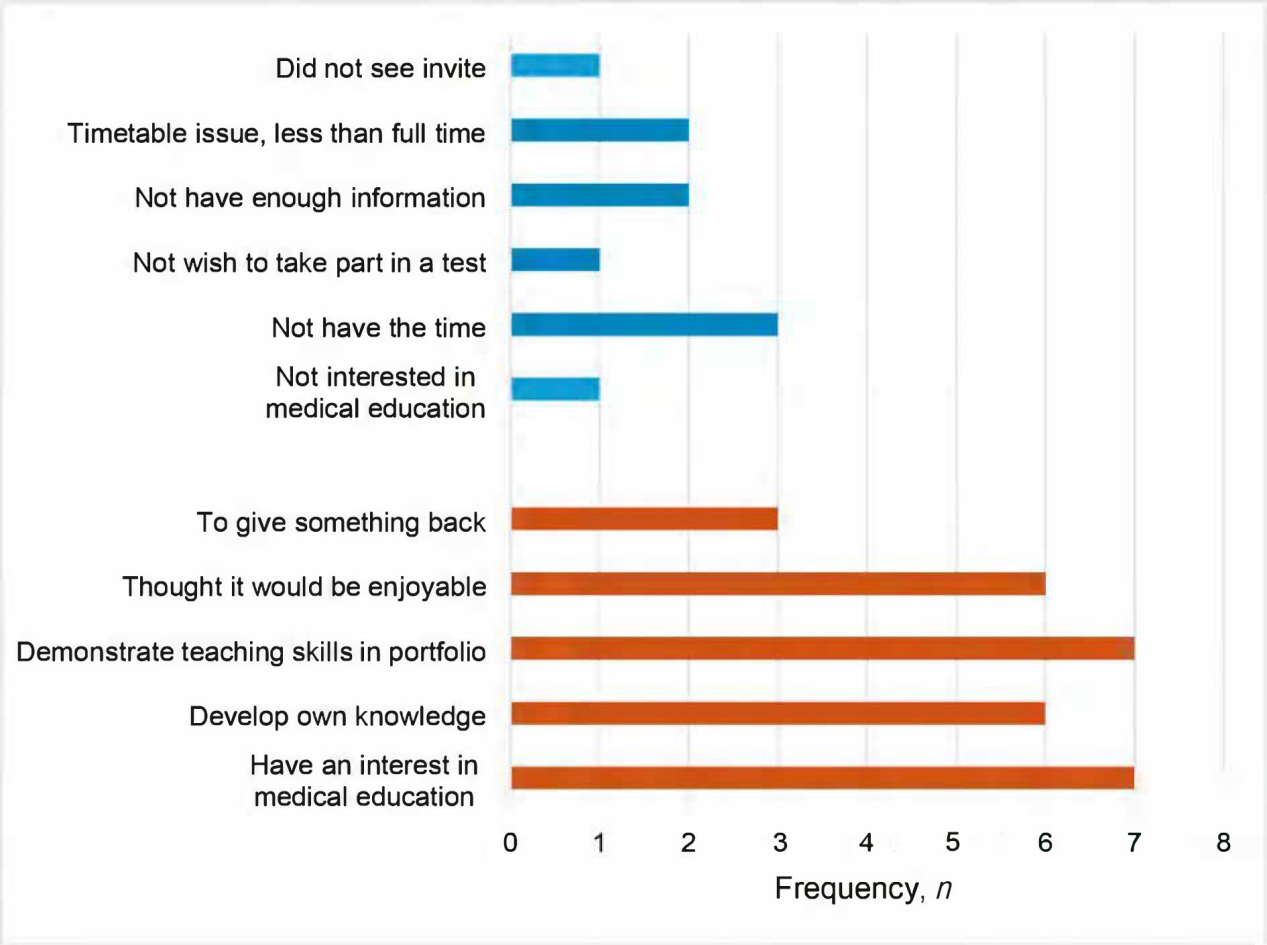
General practice specialty trainees (GPST) reasons for and against participating in near peer teaching of medical students Not participating = 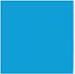 Participating = 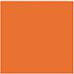 *n* = 18 GPSTs (*n* = 7 participating; *n* = 11 not participating). GPSTs provided multiple reasons for participating and not participating.

The responses from GPSTs (*n* = 18) who were invited to participate in the study are summarised in Figure 1. A total of 29 reasons for participating and 10 reasons for not participating were provided. The most common reported motivators for participating were having an interest in medical education (7/29 [24%]) and the opportunity to demonstrate teaching skills in their portfolio (7/29 [24%]). The most frequent reasons for not participating were insufficient time (3/10 [30%]); lack of timetable space (2/10 [20%]), and receiving insufficient information about the project (2/10 [20%]).

### Stakeholder views about NPT

#### Medical student views

Themes from the free-text responses of students placed with GPST and GP tutor supervisors described the GEM visits as positive learning experiences and students gaining a benefit from applying knowledge within clinical settings at an early stage of their studies. Suggested improvements focused on reducing the variation of clinical experiences between GP practices and types of patient interactions (telephone and face-to-face patient interactions) but did not identify issues relating to the supervision received.

#### GPST outcomes and views about NPT

**Figure 2.**
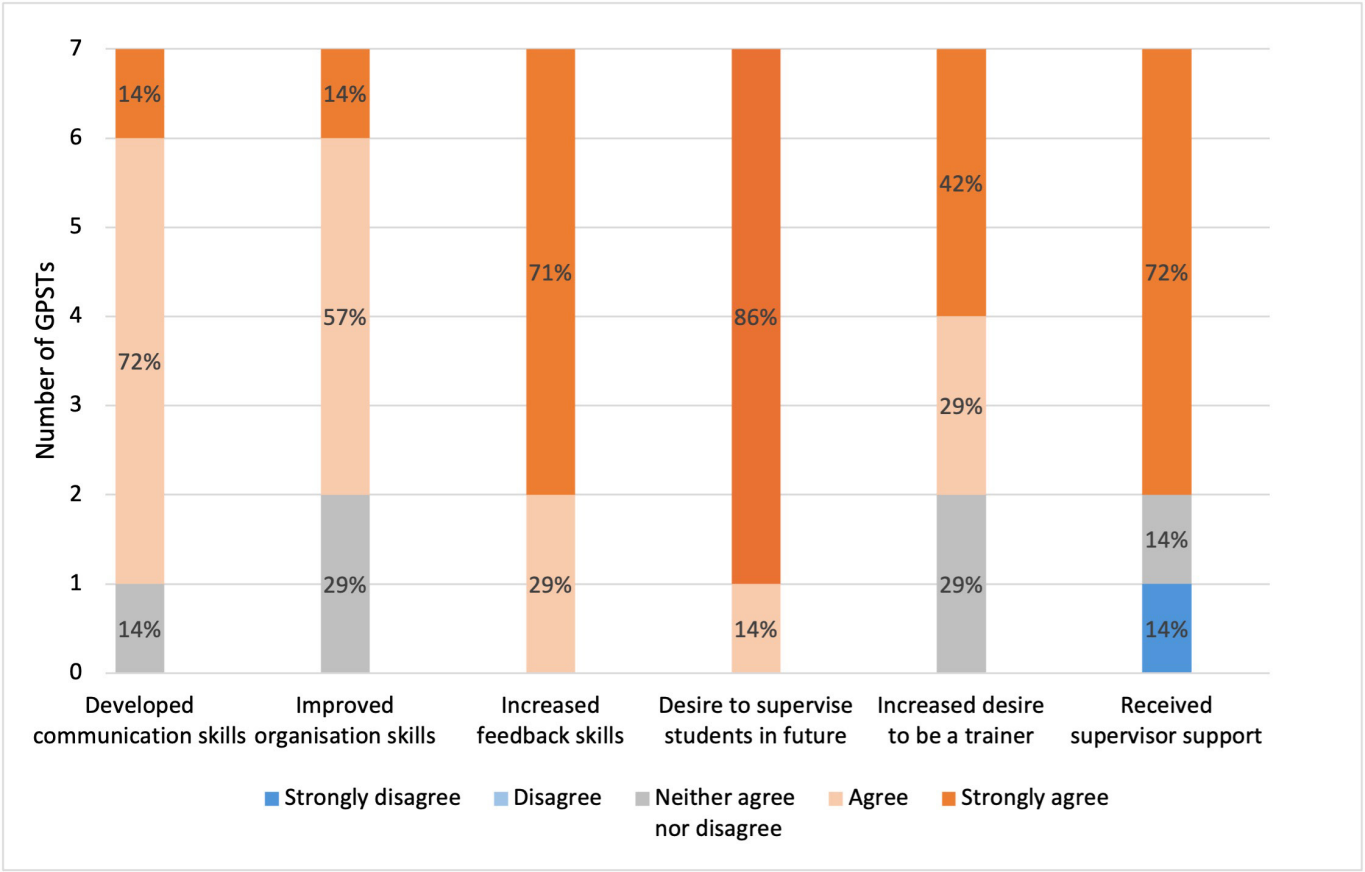
General practice specialty trainees (GPST) outcomes from near peer training (NPT)

The outcomes for GPSTs from undertaking NPT are summarised in Figure 2. All GPSTs (7/7, 100%) reported an increase in feedback skills and a desire to supervise students in the future. The majority reported improved communication (6/7, 86%), organisation skills, and an increased desire to be a trainer in the future (both 5/7, 71%). Free-text replies described NPT as an enjoyable, affirming experience. Three GPSTs reported challenges with timetabling and practice arrangements for NPT.

#### GPST trainer views

The GP trainers of the participating GPSTs (*n* = 2) reported being supportive of the NPT pilot and that NPT exposure had developed the communication and teaching skills of GPSTs. The GPST trainers also reported that NPT of medical students may not be appropriate for all GPSTs.

## Discussion

### Summary

This pilot study found that attainment of ILOs during undergraduate GP placements was at least equivalent when students were supervised by GPSTs compared with GPs. GPSTs reported opportunities for professional development as motivating them to engage with NPT and reported improved competencies in medical education.

### Strengths and limitations

This is the first study comparing the attainment of ILOs from clinical training delivered by GPSTs and qualified GPs. Additionally, this study evaluated the views of different stakeholders involved with NPT thus providing a holistic perspective to the evaluation. This strengthens the transferability of findings into routine education practice where the perspectives of multiple stakeholders are important when implementing this complex intervention. The high survey response rates from GPST and medical students improves the generalisability of findings to these target populations.

A weakness, however, was the small sample of GPSTs limiting the power to the study for statistical comparisons. Owing to small sample sizes, we were unable to adjust for potential confounders, such as age and gender, thereby introducing the possibility for residual confounding. In addition, the small numbers of participants, in particular GPSTs, at a single site impacts on the broader generalisability of findings. Transferability to other sites would be an important next step in evaluation of the merits of the scheme. The low response from GP trainers limits the representativeness of findings about their views about NPT. Finally, the primary outcome was self-reported attainment of ILOs by medical students and objective measurement of ILO attainment would have strengthened the findings.

### Comparison with existing literature

While there are no studies comparing attainment of ILOs in medical students, our study found GPSTs reporting improved feedback, communication, and organisational skills, and desire to continue future NPT, which is consistent with other studies.^12,13,16^

Previous studies have been qualitative in nature^9,11,13^ or assessing the outcomes of mentoring rather than teaching.^12,13,17,18^ Mentoring and coaching are leadership behaviours expected by General Medical Council^19^ and current GPSTs are required to evidence leadership activity within training.^20^ GP specialty training enables the development of leadership skills through training in knowledge and skills^21^ but experiential learning is also required to develop leadership competencies.^22^ The longitudinal nature of this project enabled teaching and mentoring roles to be combined and NPT could provide opportunities for leadership development in GPSTs.

A systematic review identified benefits from NPT in medical students beyond personal and professional development, highlighting the potential to support the wellbeing of early years medical students when transitioning into higher education.^17^ Other benefits, not evaluated as part of our study, could be the role modelling through NPT to promote career choices in general practice during the earlier stages of medical training.^23^

An area of concern from GP trainers is that medical students’ reduced contact with experienced GPs impacts on learning^12,24^ but other research suggests students are able to have wider discussions if placed with a near peer mentor.^18^ In our study, we found medical students taught by GPSTs rated their attainment of ILOs equally when compared with those taught by GPs. Our study found some ILOs had greater attainment with NPT, which could reflect the greater time available for teaching by GPSTs than GPs.

### Implications for research and practice

In this pilot study attainment of ILOs for GEM medical students during their early years clinical visits at GP practices was found to be at least equivalent between GPST and GP tutor supervision. Furthermore, NPT enables the professional development of GPSTs within medical education. Facilitating motivated GPSTs to become NPTs of early years medical students could support the building of supervisor capacity for undergraduate medical training in primary care.

Research is required to test the generalisability of findings across other early years medical students and evaluation of outcomes against objective measures of attainment. Furthermore, research that evaluates the transferability of findings and scalability of NPT across medical schools would help to better understand the potential of NPT for increasing capacity for training medical students in the future.

## Data

The dataset relied on in this article is available from the corresponding author on reasonable request.
